# Supplementary material for: Inhibition of Antiestrogen-Promoted Pro-Survival Autophagy and Tamoxifen Resistance in Breast Cancer through Vitamin D Receptor
Source: Nutrients. 2021 May 19;13(5):1715. doi: 10.3390/nu13051715 (PMC8159129; doi:10.3390/nu13051715)

Inhibition of antiestrogen-promoted pro-survival autophagy and tamoxifen resistance in breast cancer through vitamin D receptor

Ye Li [1], Katherine L Cook [1], Wei Yu [1], Lu Jin [1], Kerrie B Bouker [1], Robert Clarke [1] and Leena Hilakivi-Clarke [1]

[1]Department of Oncology, Georgetown University, Washington, District of Columbia.

Current address for Y Li: The University of Texas MD Anderson Cancer Center UT Health Graduate School of Biomedical Sciences, Houston, Texas; for KL Cook: Department(s) of Surgery and Cancer Biology, Wake Forest School of Medicine, Winston-Salem, North Carolina; and for Lu Jin, Robert Clarke and Leena Hilakivi-Clarke: The Hormel Institute, University of Minnesota, 801 16th Ave NE, Austin, MN 55912

Corresponding author: Leena Hilakivi-Clarke, The Hormel Institute, University of Minnesota, 801 16th Ave NE, Austin, MN 55912. Phone: (507) 437-9600. E-mail: hilak001@umn.edu.

Running title: Vitamin D receptor, autophagy and breast cancer

Supplementary Tables S1-S3

Supplementary Table S1. Datasets used to assess whether VDR expression in ER+ mammary tumors is predictive of survival and to identify signaling pathways linked to VDR expression.

Data set	Platform	Country	Cases	IHC	Adjuvant therapy	Refs.
GSE-17705	[HG-U133A] Affymetrix	USA	298	ER,PR	Tamoxifen	(31)
GSE-12093	[HG-U133A] Affymetrix	USA	136	ER	Tamoxifen	(32)
GSE-6532	[HG-U133 Plus2.0] Affymetrix	Canada	87	ER,PR	Tamoxifen	(33)
GSE-1379	Arcturus 22k microarray	USA	60	ER,PR, HER2	Tamoxifen	(34)
**Total**			**581**			

Supplementary Table S2-1. Differentially expressed genes (DEGs) involved in biofunction of cell death and survival between mammary tumors expressing above median and below median VDR.

Gene	Freque	Gene	Freque	Gene	Freque	Gene	Freque	Gene	Freque
ACTN1	2	ATG9B	1	CAMK	1	CHR	1	DLG5	1
ADAM	1	ATG13	1	CANX	1	CIDE	1	DLG	1
ADAM	3	ATP6V0	1	CAPS	1	CITE	1	DLX5	1
ADAM	1	ATP7A	1	CBX4	1	CKS2	1	DNAJ	1
ADCY1	1	ATP7B	1	CCDC8	2	CLEC	1	DNM	2
ADIPO	1	AURKA	2	CCL25	1	COL1	1	DPT	2
AK5	2	BAG4	1	CCNB1	2	COL4	1	DTL	1
ALB	1	BATF	1	CCND1	2	COL4	3	DUSP	1
ANPEP	1	BCL10	2	CCNE2	1	COL6	4	EGFR	1
ANXA	1	BCL2	2	CCNG1	1	COL6	3	EGR1	1
APOB	1	BCL2L11	3	CD164	1	COM	2	EGR3	1
APOBE	1	BCL2L13	1	CD24	2	COPS	1	EHF	2
APOD	1	BCL2L14	1	CD3E	1	COPS	1	EIF3	1
ARHG	1	BCL2L2	1	CD46	1	COPS	1	EIF5	1
ARL2B	1	BCL3	1	CD74	2	COX1	1	ELF3	1
ARRB1	1	BCL6	1	CDH1	1	COX1	1	ELN	1
ASAH1	2	BCLAF1	3	CDH2	1	CRAB	1	EMP1	1
ASCL1	3	BECN1	2	CDK1	4	CRIP1	1	EMP3	2
ASPH	2	BHLHE4	1	CDK5R	1	CSF3	1	ENC1	1
ATAD2	2	BIRC5	1	CDKN2	1	CST6	1	ENTP	1
ATF3	1	BMPR1B	2	CEAC	1	CTSZ	1	EPB4	2
ATG10	2	BNIP3	2	CEAC	2	CTTN	2	EPCA	1
ATG12	4	BNIPL	1	CELSR	1	CX3C	1	EPOR	1
ATG13	1	BRIP1	1	CENPA	1	CYP1	3	ERAL	1
ATG3	1	C3	2	CENPE	1	CYP2	2	ERBB	1
ATG4A	2	C4A	1	CERS6	2	CYR6	2	ERBB	1
ATG4B	1	C8orf44-	1	CFB	1	DCN	3	ERN1	1
ATG5	3	CA9	1	CHI3L1	4	DGKE	1	ERO1	1
ATG7	1	CALCA	1	CHMP	2	DHRS	1	ESR1	4
ATG9A	3	CALR	1	CHRM	1	DKK3	1	ESRR	1

Supplementary Table S2-2. DEGs between higher and lower VDR expression ER+ breast cancers involved in biofunction of cell death and survival.

Gene	Freque	Gene	Freque	Gene	Freque	Gene	Freque	Gene	Freque
FABP4	1	GALN	1	HSP90	1	INSM1	1	LTBP1	1
FABP5	1	GBP1	1	HSP90	1	IQGAP	1	LTBP3	1
FAIM	1	GCAT	1	HSPB1	1	ITGAL	1	LTF	3
FAM1	1	GCLC	1	HSPB8	1	ITGB2	1	LUM	1
FAS	2	GDF15	1	HSPD1	1	ITGB8	2	LYZ	1
FAST	1	GFRA1	4	HSPG2	2	ITPKC	1	MAD2L	1
FBLN	3	GHR	1	HTRA1	1	ITPR2	1	MAGE	2
FBLN	1	GJA1	1	HUWE	1	JUP	1	MAGI1	1
FBN1	4	GJB2	2	ICAM1	2	KCND	1	MAN2C	1
FEN1	1	GLG1	1	ID3	1	KCNK	1	MAOA	1
FGF18	1	GLUL	1	IFI16	1	KCNN	1	MAOB	1
FGFR1	1	GMNN	1	IFI27	1	KCTD1	1	MAP1B	1
FGFR2	1	GNAS	1	IFIH1	1	KDM3	1	MAP2K	1
FGFR3	4	GNB2L	1	IFITM1	1	KIT	1	MAPK1	1
FHL2	1	GPX3	1	IFNA1/	1	KLRC1	1	MAPK1	1
FLCN	1	GREM	1	IGF2	1	KRT14	1	MAPK1	1
FLNA	3	GRIA2	2	IGFBP3	1	KRT2	1	MAPK1	1
FN1	4	HAT1	1	IGFBP5	1	LAMB	1	MAPK8	1
FNTA	1	HDAC	1	IGHG1	1	LAMP	1	MAPK9	1
FOS	1	HDAC	1	IGHM	1	LAMT	1	MAPKA	1
FOXA	1	HERP	1	IGKC	1	LEPR	1	MAPKA	1
FOXN	2	HIGD1	1	IL11	1	LGALS	1	MAPT	2
FOXO	1	HINT1	1	IL1R1	1	LGALS	1	MB	1
FRS2	1	HIP1R	2	IL2RA	1	LGALS	1	MBD2	1
FST	1	HIST1	1	IL6ST	1	LIMK1	1	MDM2	1
FUBP1	1	HLA-	2	ILF3	1	LMO3	1	MED6	1
FXR1	1	HLA-G	1	INHBA	1	LOX	3	MEG3	1
G0S2	1	HMGB	1	INPP4	1	LOXL1	2	MEN1	1
G6PD	1	HNRN	1	INPP5	1	LOXL2	1	MICAL	1
GABR	1	HOXC	1	INSIG1	1	LPAR1	2	mir-	1

Supplementary Table S2-3. DEGs between higher and lower VDR expression ER+ breast cancers involved in biofunction of cell death and survival.

Gene	Freque	Gene	Freque	Gene	Freque	Gene	Freque	Gene	Freque
mir-	1	NR2C2	2	PIP	2	PSMD	1	RUNX	1
MMP	1	NRG1	1	PKM	1	PTGIS	1	S100A1	1
MMP	1	NTN4	1	PLAGL	1	PTK6	1	S100A4	1
MMP	3	NTS	1	PLAU	2	PTN	2	S100A6	2
MMP	3	NUF2	1	PLAU	3	PTP4A	2	S100A7	3
MMP	1	OCLN	1	PLCB1	1	PTPN1	2	S100A8	2
MMP	1	OPRM	1	PLOD2	2	PTPN1	1	S100A9	1
MNA	1	OSBPL	1	PLP1	2	PTPRC	1	SAT1	1
MRPS	3	OSMR	1	PLS3	1	PTPRT	1	SCGB3	2
MS4A	1	OVOL	1	PLXNB	1	PVRL2	2	SCN10	1
MSTN	1	PAK1	1	PMAIP	1	PXDN	1	SDC1	2
MTD	1	PAWR	1	PMEP	3	QSOX1	1	SDC2	3
MUC1	3	PAX3	1	PMP22	1	RABG	1	SDR16	1
MXD1	1	PCBP2	1	PNN	1	RAB32	1	SEC23	1
MYC	1	PDCD	1	POLR2	1	RAC2	1	SEC61	2
MYH1	1	PDCD	1	POSTN	1	RAD21	1	SERP1	1
MYL9	1	PDE4B	1	POT1	1	RAD51	1	SERPI	2
MYLK	1	PDGF	1	PPP1R	1	RAD52	1	SERPI	1
MYO1	1	PDGF	2	PPP1R	2	RB1CC	2	SERPI	1
MYO6	1	PDK4	1	PRDM	1	RBM25	1	SERPI	3
NCA	1	PDLIM	1	PRDX2	1	RBP1	11	SFRP1	2
NCAP	1	PDZK1	2	PRKA	1	RBP4	1	SFRP2	1
NCO	2	PFDN4	1	PRKA	1	RERG	1	SFRP4	2
NELL	1	PFKP	1	PRKA	1	RET	2	SIAH2	1
NFIB	2	PGK1	1	PRKA	1	RIT1	1	SIX1	1
NFIX	1	PHLD	2	PRLR	2	RNF14	1	SIX2	1
NFKB	1	PHLD	2	PRMT2	1	RPL38	1	SLC16	1
NLK	1	PIGF	1	PROM	3	RRM2	1	SLC28	1
NOV	1	PIGZ	1	PRPF4	1	RSF1	1	SLC30	1
NPY5	1	PIK3C	1	PSMD1	1	RTN1	2	SLC39	3

Supplementary Table S2-4. DEGs between higher and lower VDR expression ER+ breast cancers involved in biofunction of cell death and survival.

Gene	Frequency	Gene	Frequency	Gene	Frequency
SLC7A11	2	TFAP2B	2	TSPAN1	1
SLC7A5	1	TFPI2	1	TUBB6	1
SLC9A3R1	1	TGFB1	1	TXNL1	1
SMAD5	1	TGFB1I1	1	UBE2B	1
SMARCA1	1	TGFBR3	2	UBE2C	1
SMARCB1	1	THBS1	1	UBE2E3	1
SMARCE1	1	THY1	2	UBE2K	1
SMN1/SMN2	1	TIE1	1	UGCG	1
SMPD3	1	TIMP3	2	ULK2	1
SOCS2	1	TIMP4	1	UNC5B	1
SORBS2	1	TKT	1	USP1	1
SOX9	1	TMX1	2	USP14	1
SPARC	3	TNC	4	VAMP7	1
SPP1	1	TNFRSF11B	1	VBP1	1
SQSTM1	1	TNFRSF12A	1	VCAN	4
SRI	1	TNFRSF19	1	VTCN1	2
SRPX	3	TNFRSF25	1	WDR72	1
ST6GAL1	1	TNFSF4	1	WISP1	1
STC1	3	TNIK	2	WNT6	1
SUB1	2	TOP2A	2	WT1	2
SULF1	3	TOX3	3	WWTR1	1
SULF2	2	TP53BP1	1	XBP1	1
SUZ12	1	TP63	1	YBX3	1
TAF9	1	TPD52	3	YEATS4	1
TBC1D3H	2	TPM1	1	YLPM1	1
TBC1D9	2	TPX2	1	ZFP36L2	1
TBL1XR1	1	TRA2A	2	ZNF385B	1
TBX3	2	TRAF4	1	ZNF91	1
TCF4	2	TRIM29	1		
TDP2	1	TRIP6	1		

Supplementary Table S3. Changes in autophagy genes in TAM resistant LCC9 human breast cancer cells transfected with control (NC) or VDR siRNA, treated with TAM, VitD or their combination.

	siRNA-NC			siRNA-VDR		
	TAM*	VitD**	TAM*+VitD**	TAM*	VitD**	TAM*+VitD**
IRE1α	↑	↓	↓	↓	↓	↓
pIRE1α/IRE1α	↑	↓	↓	↑	↑	↑
JNK	NA	↓	↓	↑	↑	↑
pJNK/JNK	↑	↓	↓	↑	↑	↑
Bcl-2	↓	NA	NA	NA	NA	NA
pBcl-2/Bcl-2	↑	↓	↓	↑	↑	↑
Beclin 1	↑	↓	↓	↑	↑	↑
Bcl xL	NA	↑	↓	↑	↑	↑
ATG 7	↑	↓	↓	↑	↑	↑
ATG 5	↑	↓	↓	NA	NA	NA
P62	↓	↑	↑	↑	↑	↑
LC 3BII	↑	↓	↓	↑	↑	↑
VDR	NA	↑	↑	↓	↓	↓

* indicates 4-hydroxy Tamoxifen (100nM)

** indicates Calcitriol (100nM) and/or EB1089 (100nM)

NA = not altered

Red arrows indicate changes to opposite direction, compared with siRNA-NC cells

Supplementary Figures S1-5.

Figure S1. Gene enrichment analysis of VDR related differentially expressed genes (DEGs) was conducted using Ingenuity Pathway Analysis (IPA) showing (**A**) top 20 bio-functions and (**B**) top 20 canonical pathways.

A.

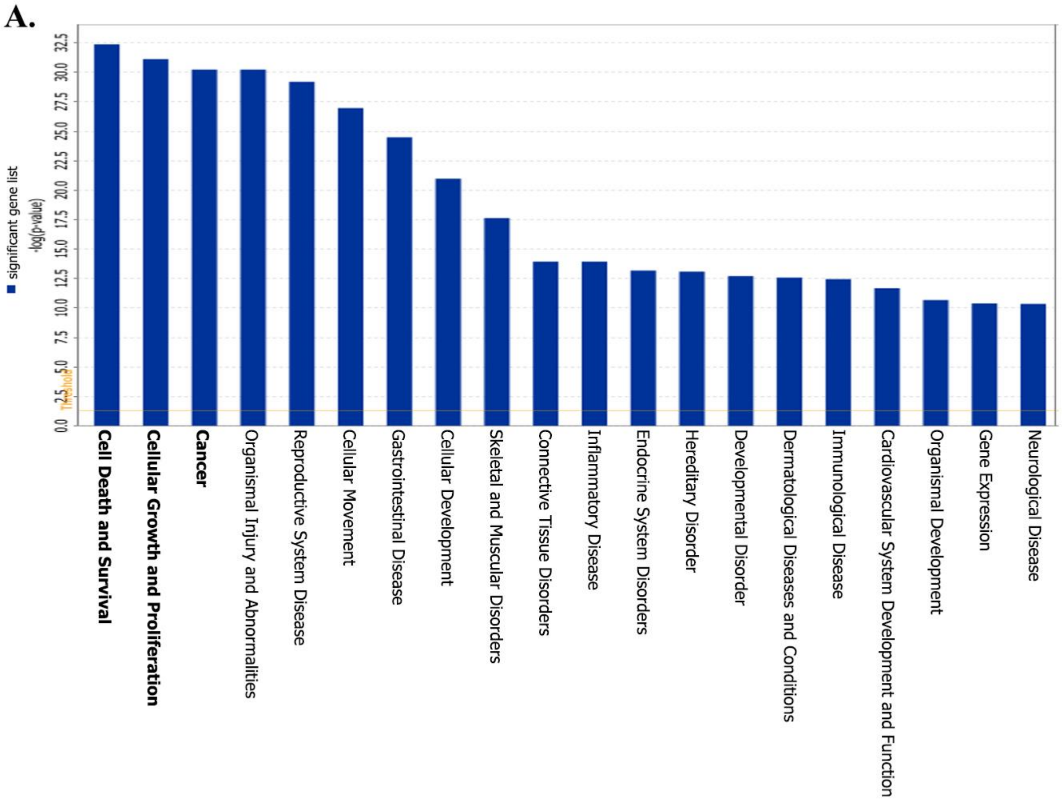

B.

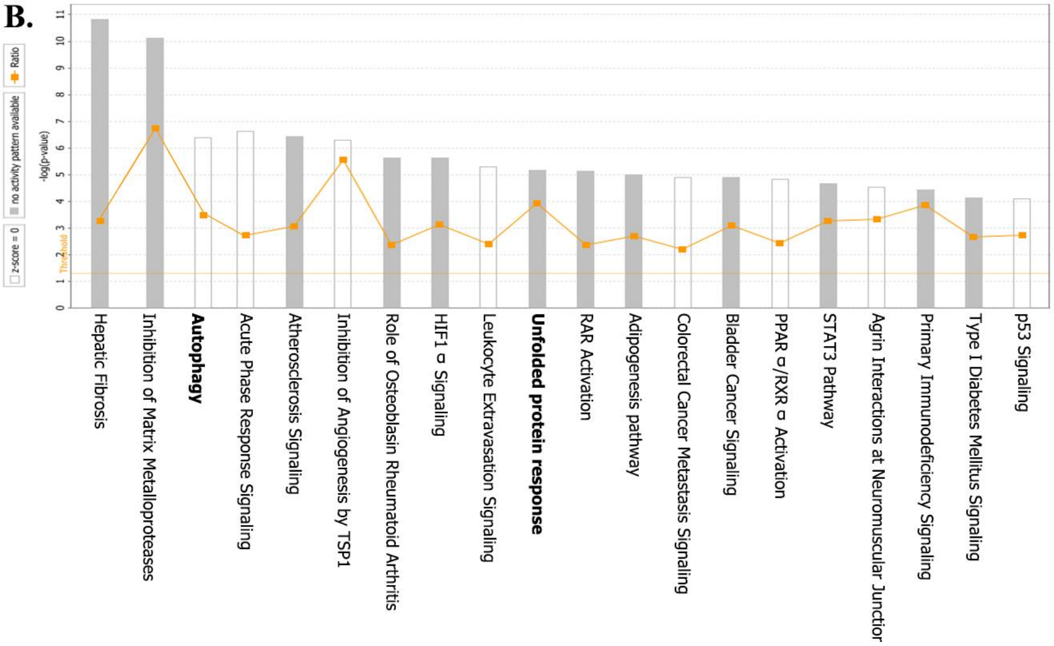

Figure S2. Calcitriol and EB1089 inhibited the activation of IRE1α-JNK signaling pathway in breast cancer cells. Quantification of western blot analyses of (**A**) VDR, (**B**) Beclin 1, (**C**) Atg7, (**D**) LC3BI, (**E**) LC3BII, (**F**) p62 in TAM sensitive LCC1 and TAM resistant LCC9 breast cancer cell lines. Graphs represent three independent biological replicates. **P*<0.05, NS: not significant. Means ± standard error of means are shown.

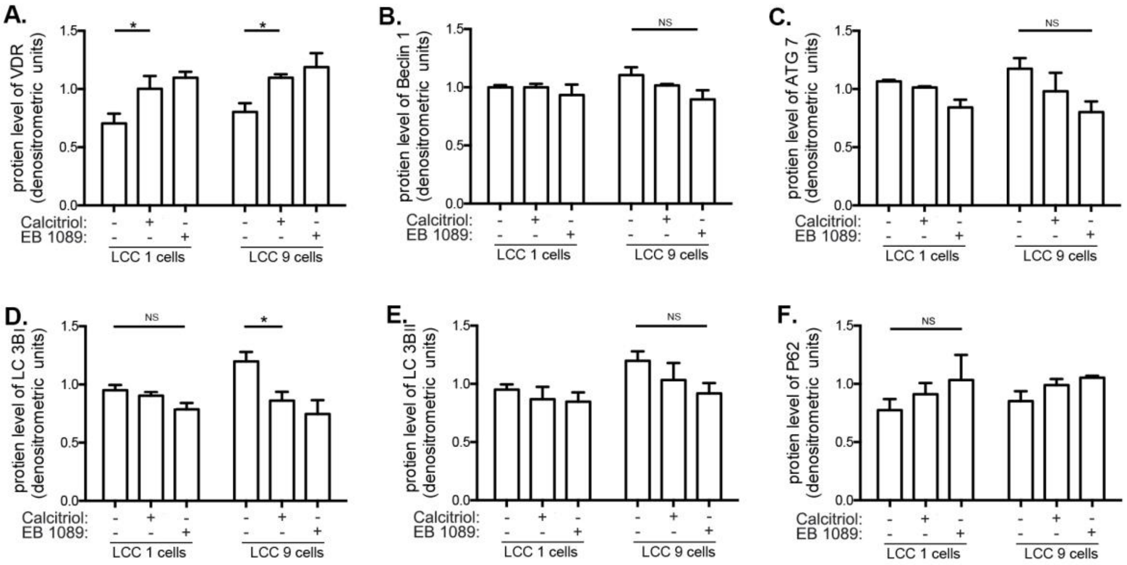

Figure S3. Calcitriol and EB1089 enhanced and restored response of breast cancer cells to TAM treatment. Quantification of western blot analyses of (**A**) VDR, (**B**) IRE-1α, (**C**) the ratio of pIRE-1α over total IRE-1α, (**D**) total JNK, (**E**) the ratio of pJNK over total JNK, (**F**) LC3BI in TAM sensitive LCC1 and TAM resistant LCC9 breast cancer cell lines. Quantification is based upon three independent biological replicates. **P*<0.05, NS: not significant. Means ± standard error of means are shown.

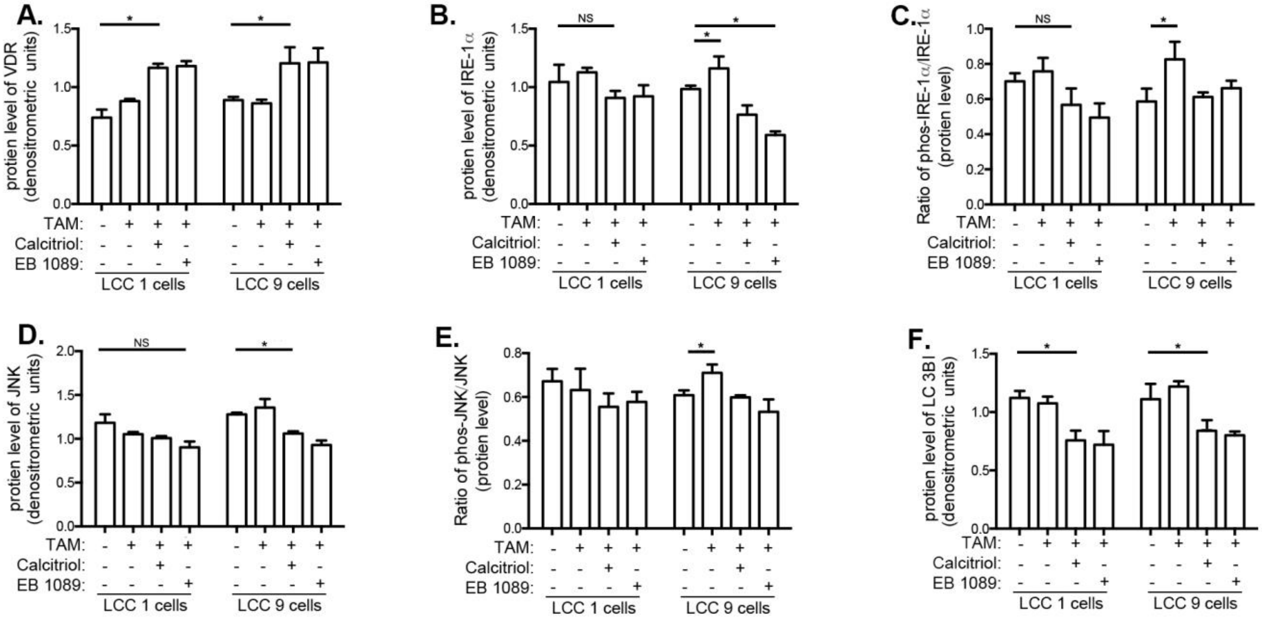

Figure S4. Silencing VDR inhibits VitD action on pro-survival autophagy genes in breast cancer cells. Quantification of western blot analyses of (**A**) VDR, (**B**) Atg7, (**C**) LC3BI, (**D**) LC 3BII, and (**E**) p62 in TAM sensitive LCC1 and TAM resistant LCC9 breast cancer cell lines. Quantification is based upon three independent biological replicates. **P*<0.05; means ± standard error of means are shown.

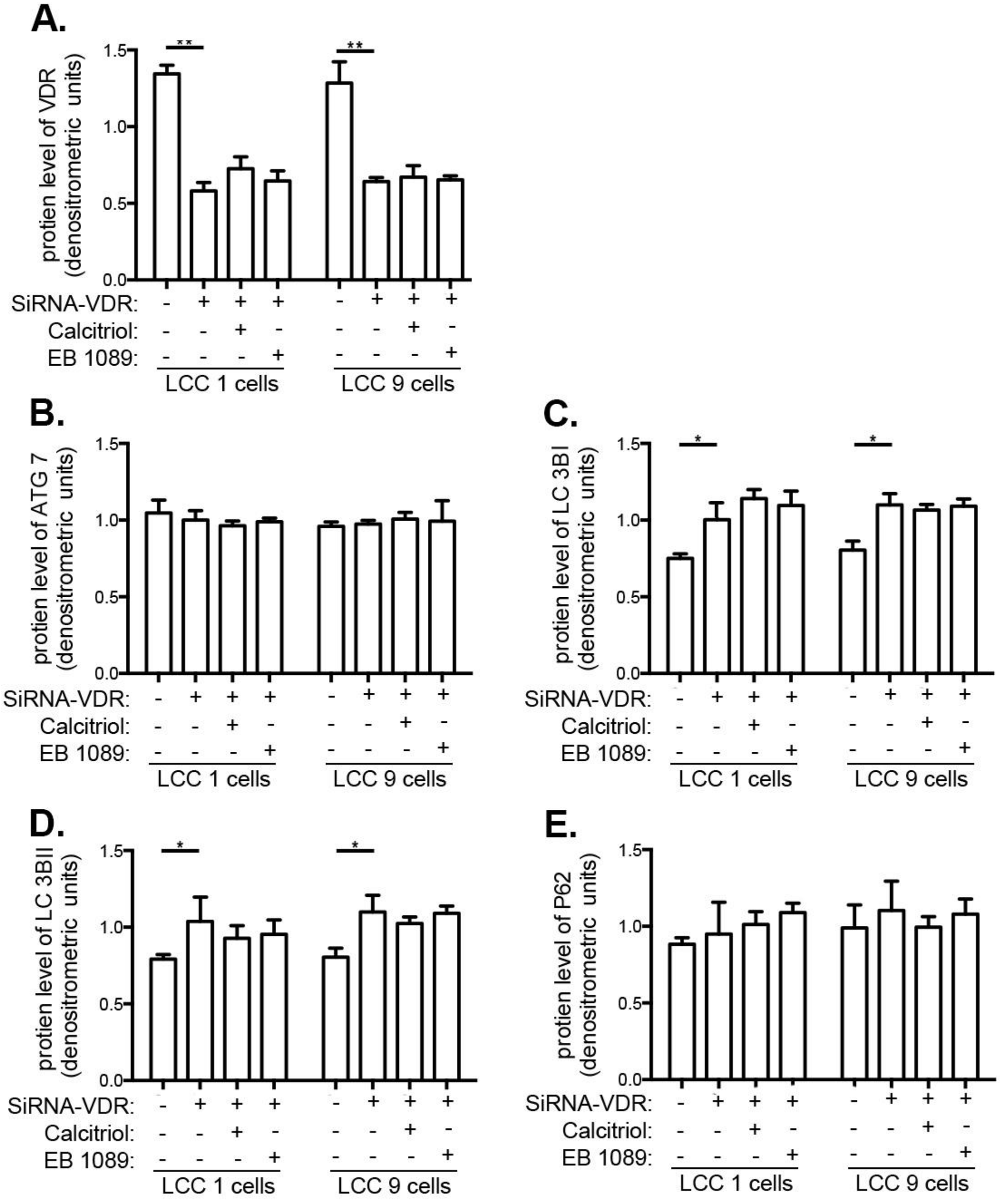

Figure S5. Silencing VDR prevents pro-survival autophagy response of breast cancer cells to VitD and TAM treatment. Quantification of western blot analyses of (**A**) VDR, (**B**) total IRE-1α, (**C**) the ratio of pIRE-1α over total IRE-1α, (**D**) total JNK, (**E**) the ratio of pJNK over total JNK, and (**F**) LC3BI in TAM sensitive LCC1 and TAM resistant LCC9 cells. Quantification is based upon three independent biological replicates. *P<0.05, ** P<0.01. Means ± standard error of means are shown.

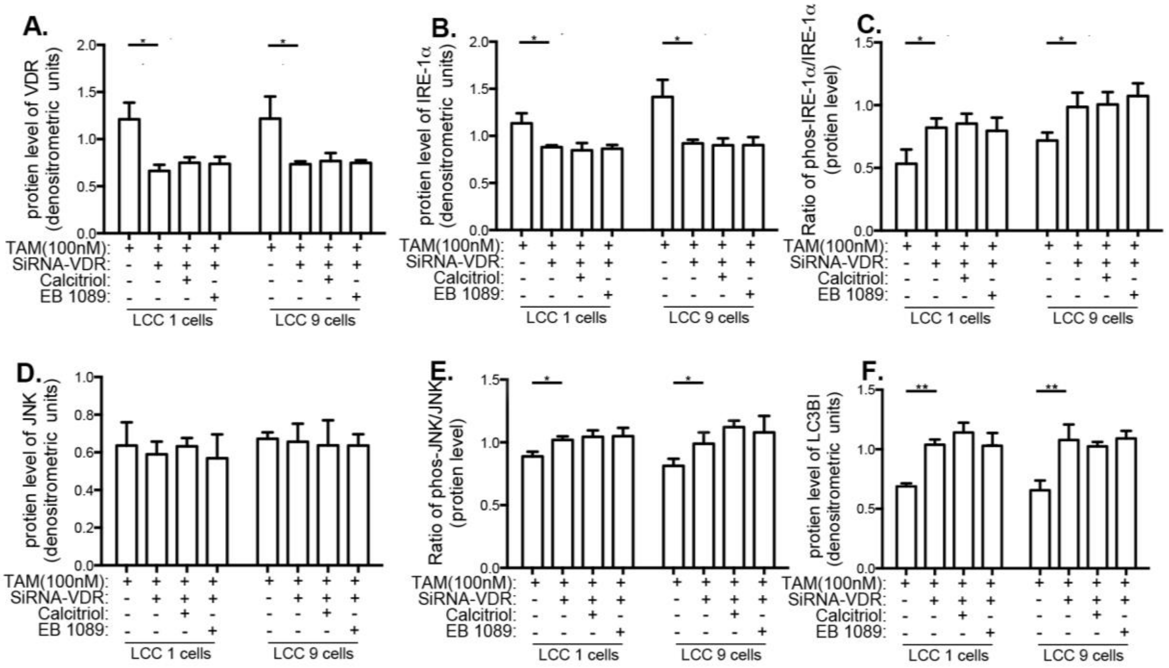

Supplement: Supplementary file 1 [file nutrients-13-01715-s001.zip › nutrients-1196445-supplementary.pdf]
